# Asymmetric flows and drivers of herbaceous plant invasion success among Mediterranean-climate regions

**DOI:** 10.1038/s41598-018-35294-7

**Published:** 2018-11-15

**Authors:** Miguel A. Casado, Irene Martín-Forés, Isabel Castro, José M. de Miguel, Belén Acosta-Gallo

**Affiliations:** 1 Ecology, Department of Biodiversity, Ecology and Evolution, Complutense University of Madrid, 28040 Madrid, Spain; 2 Department of Biogeography and Global Change, National Museum of Natural Sciences, Spanish National Research Council, 28006 Madrid, Spain; 3 Department of Ecology, Autonomous University of Madrid, 28049 Madrid, Spain

## Abstract

Understanding the mechanisms that support the arrival, establishment and spread of species over an introduced range is crucial in invasion ecology. We analysed the unintentionally introduced herbaceous species that are naturalised in the five Mediterranean-climate regions. There is an asymmetry in the species flows among regions, being the Iberian Peninsula the main donor to the other regions. At interregional scale, the species’ capacity to spread among regions is related to the ecological versatility of the species in the donor area (Iberian Peninsula). At intraregional scale, the species’ capacity to successfully occupy a complete region first depends on the time elapsed from its introduction and afterwards on the degree of occurrence in the region of origin, which is commonly related to its chance of coming into contact with humans. Information on exotic species in their origin region provides insights into invasion process and decision-making to reduce the risks of future invasions.

## Introduction

The geographical transport of species associated with human activity has increased with time, currently presenting unprecedented levels^1^. This process is responsible for over 13,000 plant species having become naturalised outside their native range^1^, a fact that has high economic and ecological costs^2^. Likewise, numerous studies have highlighted their adverse effects upon biodiversity and ecosystem functioning^3,4^. Ascertaining the mechanisms promoting the arrival, establishment and spread of exotic species is therefore the central focus in the study of invasion ecology^5–7^.

Many species attributes, such as life history, performance-related traits, original range and biogeographic origin have been analysed as predictors of the success of exotic plants^8–11^. However, very few of these characteristics have revealed consistent patterns^12^, and their capacity for prediction has therefore been questioned^6,8,13,14^. Likewise, extrinsic factors such as environmental heterogeneity, time since introduction^5^, propagule pressure^15,16^ or human disturbance^17,18^ have been proposed to explain the geographical extent of plant invaders. Although some of these factors have provided strongly predictive models, their usefulness has been shown to be context-, species- and scale-dependent^6,8,13,19^.

The manner in which species are transported from their native range to a new region (introduction pathways) greatly determines their invasion success. The factors influencing this success are different for deliberately introduced species (e.g. species intended for commercial use) compared with the unintentionally introduced ones^20^. Asymmetries in species flows between countries or regions may be associated with historical^21^, commercial^22^ or economic^23^ factors. These factors are particularly important in the case of deliberate introductions (release into nature, escape from confinement) where human being is not only responsible for their transport, but also provides assistance for them to become established and to spread^24,25^. Asymmetries in species flows can also occur among different subsets of species, often because given life forms are associated with one specific introduction pathway.

Numerous articles have highlighted the importance of conducting comparative studies in different regions of the planet in order to provide generalizable patterns in relation to invasion^26,27^. In particular, the Mediterranean-climate regions (MCRs) have been highlighted as being especially appropriate for these studies, given the high degree of climatic similarity, which reduces large-scale environmental variation^27–31^. Moreover, the MCRs have been, and still are, subjected to intense human transformation, and they present a large amount of exotic species^26,30,32,33^; there is therefore a vital need to comprehend the invasion processes in these regions^27,29,34,35^. In this sense, Arianoutsou *et al*.^30^ have recently compared the exotic flora of the five MCRs from different points of view: taxonomic composition, origin of the species and life-history traits. However, little is yet known about the species flow that has occurred among these regions, as well as the effect that the characteristics of these species in their region of origin have on their invasiveness. On the other hand, quantifying not only the number of naturalized exotic species but also their potential for expansion at the regional level (potential regional spread) can more clearly shed light on the processes of plant invasion at the global level.

In the present paper we analyse the patterns of distribution of exotic plant species in the Earth’s different MCRs (namely, Iberian Peninsula as a representation of the Mediterranean Basin, California, Central Chile, Western Cape and Southern and Western Australia). According to Prinzing *et al*.^9^ the term ‘exotic’ comprises all casual, naturalised or invasive species in a given region. In order to reduce the factors that may have influenced the invasiveness of the species, we have only considered the unintentional pathway species, which represent more than 80% of the exotic species in MCRs^30^. Likewise, we have only considered exotic herbaceous species because they constitute the dominant life form among the exotic plants within the Mediterranean Biome^30^; additionally, they can exhibit a different response to the colonisation process rather than that of woody species^36^. Our objectives are: (1) to analyse the degree of invasion presented by each of the five MCRs; (2) to establish the species flow among the different regions and (3) to ascertain the factors determining the invasion success of exotic species coming from the Iberian Peninsula at intraregional and interregional scale. Among the factors that determine the invasion success, we analyse the effect of some extrinsic (e.g. minimum residence time) and intrinsic (e.g. life cycle, geographical range in the donor area) characteristics of the unintentionally introduced herbaceous species. Following Lloret *et al*.^13^ we assume that when comparing climatically similar territories, invasiveness of a species would depend more on its own attributes than on the environmental characteristics of the recipient area. Under this assumption, we attempt to ascertain why within a regional species pool, some of them have succeeded in becoming naturalised and others have not. In this sense, the obtained results will provide a better understanding of species’ naturalisation patterns and will help to identify their potential for invasion prior to their arrival in a new region^9,13,19^.

## Results

### Flows among regions

All five MCRs included a total of 1,934 naturalised herbaceous species, most of these (1,125) species native to one of the five regions studied (Table 1). Most of the naturalised species (73%) corresponded to unintentionally introduced species, followed by ornamental ones (21%) and crops (6%). The regions of California and S-W Australia were noteworthy, with over 1,000 naturalised species (approximately 75% unintentional), whereas Western Cape only presented 319 naturalised species (91% unintentional). In the case of species introduced for ornamental purposes (408 species), S-W Australia constituted a notable recipient area (263 species) and Western Cape stood out as a donor (146 species). As for the crops (108 species), the Iberian Peninsula was both a notable recipient area (82 species) and a donor (11 species).

**Table 1:** Number of naturalised species per Mediterranean climate regions according to introduction pathway: ornamental, crop or unintentional.

	Iberian Peninsula	California	Central Chile	S-W Australia	Western Cape	Total
Ornamental	189 (28)	193 (6)	38 (9)	263 (3)	18 (146)	408 (181)
Crop	82 (11)	57 (2)	25 (1)	58 (0)	11 (5)	108 (19)
Unintentional	250 (739)	834 (49)	450 (39)	806 (32)	290 (71)	1418 (925)
Total	521 (853)	1084 (59)	513 (52)	1127 (43)	319 (224)	1934 (1125)

In each case the number of naturalised species (i.e. MCR as a recipient area) is shown and, in brackets, the number of native species (i.e. MCR as a donor area).

**Figure 1:**
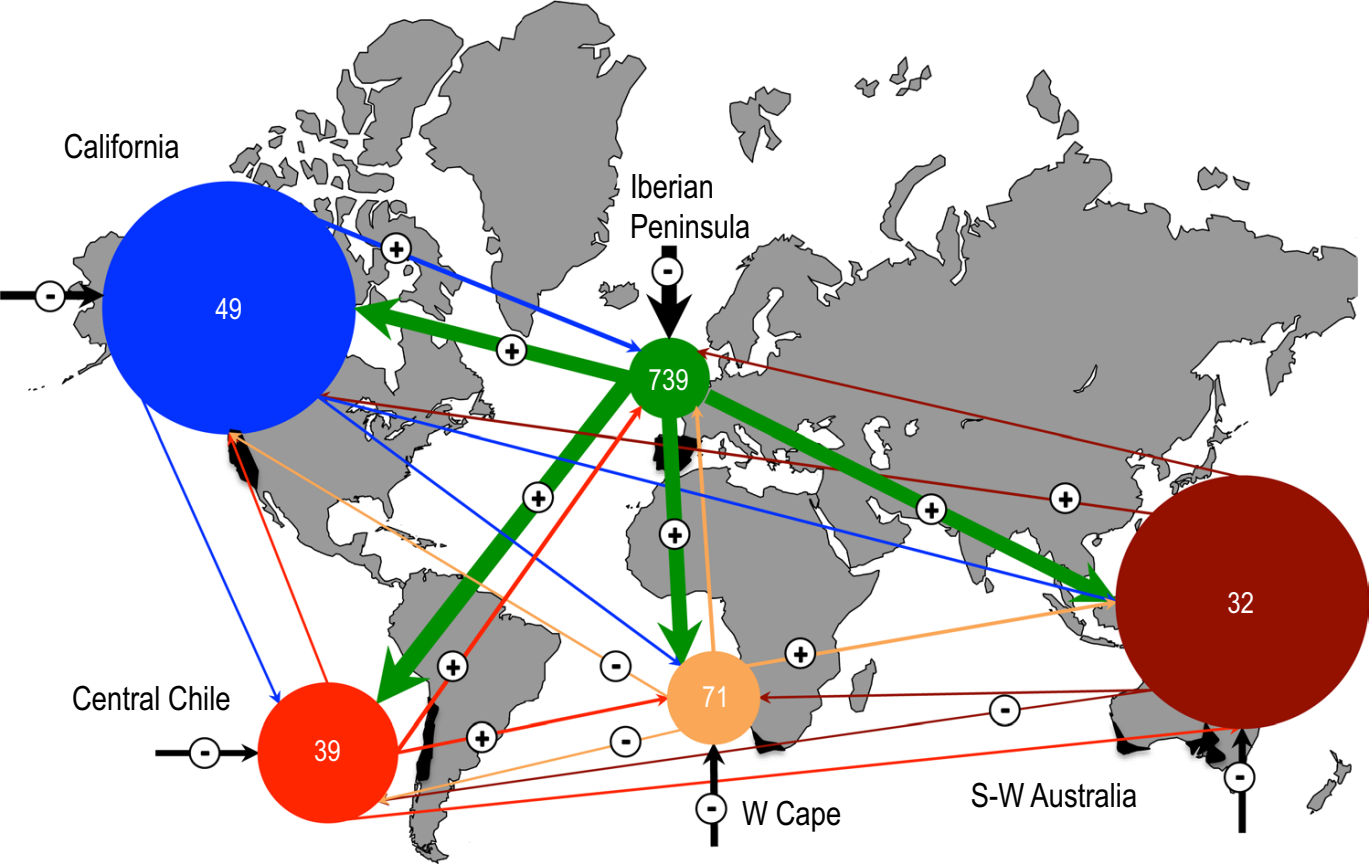
Flows of naturalised herbaceous species among the five Mediterranean regions. Only species introduced by the unintentional pathway have been considered. Each region is represented by a specifically coloured circle, the radius of which is proportional to the number of naturalised species it presents as recipient (see Table 1). The values inside the circle indicate the number of naturalised species that are native to that region, but naturalised in at least one of the other regions. The arrows represent the flows, and their thickness is proportional to the percentage of species that the donor region provides to the set of naturalised species of the recipient region. Black arrows indicate flows from regions other than the five Mediterranean ones. Positive or negative sign above the arrows respectively indicates major or minor flows rather than those expected following a randomness test of 999 simulations.

For the unintentionally introduced species, the flows among regions were highly asymmetrical (Fig. 1). In all regions, most of the naturalised species (between 70 and 80%) corresponded to species present as natives in one of the other MCRs. The only exception was the Iberian Peninsula, where 63% of its naturalised species were not native to the other MCRs studied, but rather fundamentally to America (64 species) and the western Mediterranean Basin (44 species). The Iberian Peninsula was the principal donor in all cases, with flows towards the other regions that were always above those expected at random (Supplementary Fig. 1) and in all cases providing over 60% of the naturalised species of each region (Fig. 1). From the other four regions, the flows from California and Chile to the Iberian Peninsula, from Chile to Western Cape, from Western Cape to S-W Australia and from S-W Australia to California provided more species than those expected at random. On the contrary, the flows from S-W Australia and Western Cape to Chile or from Western Cape to California provided values below those expected at random (Supplementary Fig. 1).

### Factors determining invasion success

The Iberian Peninsula is the main donor of species towards the other MCRs, so the invasion success has been analysed only for the species coming from this region. To this end 12,573 floristic relevés made in the Iberian Peninsula were considered. These relevés included 2,664 species native to the Iberian Peninsula which were selected for subsequent analyses. Of this set of species 681 appeared as naturalised in at least one of the other four MCRs with 41% (279 species) present in one single MCR and 17% (115 species) present in the other four.

**Figure 2:**
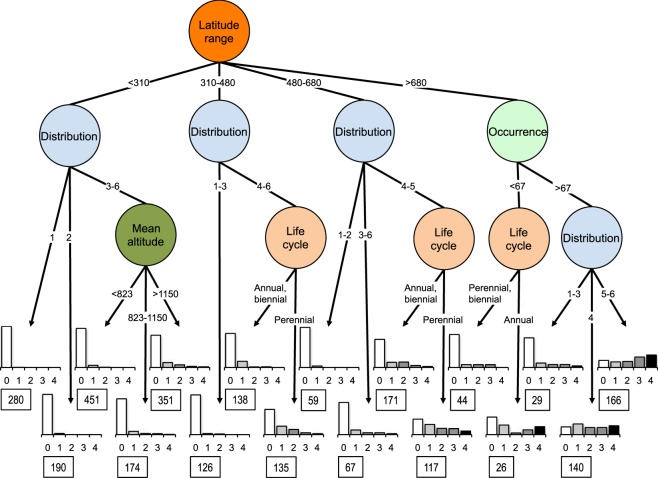
Classification tree for interregional invasion success (number of Mediterranean-climate regions in which species native to the Iberian Peninsula are naturalised) and different descriptors of the species. We applied the analysis to 2,664 species native to the Iberian Peninsula, classifying their success into five categories (graphs at the bottom): species exclusive to the Iberian Peninsula (value 0) or appearing as naturalised species in 1, 2, 3 or 4 of the other Mediterranean regions. Bar charts represent the observed frequency of interregional invasion success for the species assigned to each terminal node. The number of species assigned to each terminal node is indicated below each bar chart. For each split the cut value of the variable selected is shown. Latitude range (km) and mean altitude (m) correspond to geographic variables calculated according to the data on the Iberian relevés in which the species are present. Risk estimated value: 0.254. Occurrence is the number of relevés in which the species appears. Distribution represents the extent of the distribution area, categorised from value 1 (endemic to Iberia) to value 6 (cosmopolitan).

The classification tree for the interregional invasion success (i.e. the number of MCRs excluding the Iberian Peninsula in which a species appeared as naturalised) provided on average 75% of correct classifications associated especially with categories 0 (Iberian species not naturalised in other MCRs) and 4 (Iberian species naturalised in the 4 MCRs). The descriptor that best discriminated interregional invasion success (i. e. the number of MCRs excluding the Iberian Peninsula in which a species appeared as naturalised) was latitudinal range in the region of origin (Iberian Peninsula), followed by extent of geographical distribution area and number of occurrences in the relevés (Fig. 2). The species exhibiting less interregional invasion success were the ones presenting a smaller latitudinal range and a distribution endemic to the Iberian Peninsula: of the 280 species that met these requirements 279 have not colonised any of the other MCRs. On the contrary, the species showing greater interregional invasion success were the ones presenting greater latitudinal range in the Iberian Peninsula, with a higher frequency of occurrence in the relevés and a Eurasian or cosmopolitan geographic distribution. Of the 164 species that met these requirements 83% were colonisers of at least one MCR and 30% of the four MCRs. Only 28 of these 164 species from the Iberian Peninsula have not been naturalised in any of the other four MCRs (Supplementary Table 1) although two are present in South Africa, twelve in Australia, two in Chile, and 18 in the USA but in all cases outside of their respective MCRs. Notable in the USA are *Molinia caerulea*, *Clinopodium vulgare* and *Epilobium hirsutum*, present in states adjacent to California, *Anthyllis vulneraria*, *Juncus inflexus* and *Deschampsia flexuosa*, with old citations on the E coast and whose westward distribution has increased over time, or *Ranunculus bulbosus*, with three citations in California in 1899, 1938 and 1975, and considered to represent a failed colonisation in the present study.

**Figure 3:**
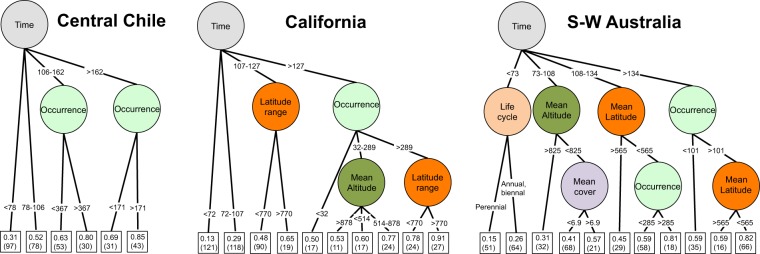
Regression trees for intraregional invasion success (percentage of territorial units within each Mediterranean-climate region where a species native to the Iberian Peninsula is naturalised) and different descriptors of the species. We applied this analysis to the species native to the Iberian Peninsula that are naturalised in each of the three Mediterranean-climate regions (Western Cape was not considered because of the lack of data available to accurately calculate intraregional invasion success). The squares at the bottom indicate invasion success (average proportion of territorial units occupied by the species in relation to the total number of territorial units in each region) and the number of species in the group appears in brackets. For each split the cut value of the variable selected is shown. Time refers to minimum residence time. The variables for latitude (in km), altitude (m) and cover (%) were calculated according to the data on the Iberian relevés in which the species is present. Occurrence is the number of relevés in which the species appears. Risk estimated value: Central Chile 0.035, California 0.053, Australia = 0.049.

Western Cape was not considered in the analysis of intraregional invasion success (i. e. the percentage of territorial units inside each MCR where a species was present) due to the lack of distribution data for exotic species inside this MCR. Time of residence was the descriptor that determined intraregional invasion success in the first place in the three MCRs, with the highest levels of success for the species with over 100 years of residence (Fig. 3). For California, Central Chile and S-W Australia the descriptors that best discriminated after time of residence were very similar: success increased with a high occurrence of the species in the region of origin and reached values higher than 80% occupation of the available range when on the Iberian Peninsula they occupied an average latitudinal range greater than 770 km (for the naturalised species of California) or an average latitude corresponding to values below around 40°30′N (in the case of S-W Australia).

## Discussion

In the set of five MCRs we found 1,934 naturalised herbaceous species. Unintentionally introduced species represent three quarters of the total, whereas those introduced for ornamental and crop purposes only represented one fifth of the total. These proportions are different from those provided by other authors, who pointed out that horticultural activity is the largest source of naturalised plants^20,31,37–39^. The differences might be related to the types of growth form considered: in our study we have excluded woody species, most of whom have been introduced for horticultural and forestry purposes^20,40^. The differences found between our results and those of other authors in relation to the number of introductions of crop, ornamental and unintentionally introduced species (Table 1) highlight the behaviour patterns of different groups of species, as well as the importance of establishing the selection criteria for this kind of studies. We chose to work only with herbaceous species that had been unintentionally introduced (i.e. which had not been deliberately disseminated by humans). Although this introduction pathway includes many types that could affect the invasiveness of a species, its choice allows us to control, at least partially, the factors associated with human interest in the species itself^8^.

Considering the unintentionally introduced naturalised herbaceous species, the lowest value occurs in the Iberian Peninsula, whereas the highest are detected in California and S-W Australia. On the contrary, the principal species donor is the Iberian Peninsula and the regions providing the lowest number of species to the remaining MCRs are S-W Australia and Central Chile. These differences cannot be attributed to the surface area of each region because, with the exception of Western Cape, and to a lesser extent Central Chile, the areas are quite similar (Supplementary Table 2). Likewise, they can neither be attributed to differences in current human population density because this variable reaches the highest and lowest values in California and Australia, respectively (Supplementary Table 2).

In order to better comprehend the outcomes of this study, it is necessary to fully understand the historical context. In the past, associated with the beginning of agriculture, the Iberian Peninsula was the recipient of exotic species although it is not easy to know the magnitude of this arrival of species (Supplementary Note 1). These archaeophytes (species introduced before the year 1500) have not been considered in this work due to the difficulty of differentiating between them and native species. Even so, of the five MCRs, the Mediterranean Basin is considered to be the most resistant to plant naturalisation^30^. This resistance has been linked to the millenary adaptation of European plants since the advent of agriculture, which have come to occupy the vacant niches, and to the poorer adaptation of extra-European species to the anthropically modified environment due to their shorter evolutionary history^1^. These historical/evolutionary considerations therefore mean that the Iberian Peninsula has the lowest values of unintentionally introduced naturalised herbaceous species.

In any case, at global scale, the set of MCRs is seen to constitute a system that is relatively closed to species exchange, with very few species entries from other regions, except in the case of the Iberian Peninsula, where most of its naturalised species are of non-Mediterranean origin. Moreover, there is a high degree of asymmetry between the main donor, the Iberian Peninsula and the four recipient areas, supplied mainly (by over 60% of their respective naturalised species) by this donor. The degree of asymmetry is even greater than the 47% provided by Dodd *et al*.^38^ as the European contribution to Australia’s exotic flora. It has recently been suggested that the traditional dichotomy Old World *versus* New World in species flows^41,42^ should be substituted by the dichotomy Northern Hemisphere *versus* Southern Hemisphere to explain the role of the continents as donors at the global scale^1,43^. At the biome scale of our study, however, this does not seem to be the case, as all the MCRs stand out as recipients and not donors, with the exception of the Iberian Peninsula. These asymmetries reflect the involvement of certain environmental characteristics (e.g. the millenary human action in the Mediterranean Basin), commercial connections, as well as, at least for Chile and California, the colonial history with the Iberian Peninsula^1,44^.

Although quantitatively most Mediterranean naturalised species come from the Iberian Peninsula, other flows are seen to present higher probabilities than those expected at random. In the first place California and Chile act as sources of species flow to the Iberian Peninsula. Both Chile and California were colonised by the Spanish, upon whom they depended until the 19th century (Supplementary Note 1). Although there was an intense exchange between Chile and California during the Californian Gold Rush^45^, these relationships were not maintained in time and do not appear to have played a decisive role in the flows of species between both regions. In the second place, Western Cape provides a flow of species to S-W Australia greater than that expected at random, and neither of these two regions have constituted a significant supply of species to the Iberian Peninsula. Both Australia and South Africa were colonised by northern Europeans (Supplementary Note 1). The Mediterranean component of their respective exotic floras not only arrived from indirect British and Dutch sources, but also through the spread of agriculture (wheat, vines) and livestock farming (sheep), directly connected with Mediterranean countries^46^. In fact, the Spanish ports of Malaga, Seville and Cadiz, as well as the Portuguese port of Lisbon, were frequented by the British and Dutch on the trips to their colonies^47^. Until the Suez Canal was opened in 1869, trade with Australia involved sailing around the Cape of Good Hope with a stopover in South African ports, a fact which might account for many of the earlier introductions^48^, as well as for the asymmetry between both regions in favour of South Africa as a donor. Thirdly, we detected a greater than expected at random flow from S-W Australia to California, likely initiated by trading during the Gold Rushes of California in 1848 and Australia in 1867 (Supplementary Note 1 and Supplementary Fig. 4) and maintained up to the present time because California is home to some of the major container ports among the MCRs^49^.

The success of Iberian species in the interregional invasion fundamentally depends upon their distribution range at different scales in their area of origin, a fact that is coherent with the results of numerous studies^6,8,9,12^. Other species attributes, such as life cycle, are hierarchically much less important, a fact that tallies with the indications of other authors^5,8,13,14^. The highest level of interregional invasion success occurs when at its origin the species presents a broad latitudinal range on the Iberian Peninsula, a high degree of occurrence at the regional scale and a very broad geographic distribution area.

These geographic variables appear to indicate the existence of two processes, not mutually exclusive, which favour interregional invasion success. On one hand, the extent both of a species’ latitudinal range and its distribution area reflects the invasion success of a species capable of living within a wide range of environments and indicates that the species has a high level of tolerance or ecological versatility^12,37^. Ecological versatility is often associated with high phenotypic plasticity^50^, so these species are more likely to survive and reproduce under different conditions in the new area^51^ and therefore have a better chance of naturalisation in a recipient region^9^. Furthermore, within the set of species presenting a broader geographic distribution, invasion success increases with occurrence at regional scale. Highly frequent species are more likely to come into contact with the vector of dispersal, i.e. human being, and therefore have a better chance of being introduced to an alien continent than rare species^9,52^.

Species’ distribution ranges in the area of origin are good predictors of interregional invasion success. However, under the prerequisites for high success (i.e. a high latitudinal range, high occurrence and an extensive geographic distribution) 28 species are included that are not present in any of the four MCRs (Supplementary Table 1). These are mostly species already present in the respective country, although not in the Mediterranean region considered. This set of species covers a wide variety of ecological situations in the Iberian Peninsula, from the most arid Mediterranean habitats to those of environments with less water stress. Several reasons could explain why these species are not naturalised in the MCRs: i) they have not yet been introduced into the MCR and it is only a matter of time, ii) they have not reached the sites that are suitable for its establishment, or iii) they are less well adapted to climatic conditions different from those of its region of origin. We therefore consider that these erroneous classifications do not invalidate the model obtained in our study, but rather reflect the need to consider other variables related to the time lag until they become effectively naturalised^53^ or propagule pressure. In any case, in the context of invasion prevention, these specific species should be subjected to a special follow-up, given their potential to colonise the MCRs.

In this study we have used the range of species within each MCR as an estimate of their intraregional invasion success. The extent of the distribution of a species may be associated with characteristics of the species that favour its spread but also with external factors, such as the existence of multiple introduction events within the MCR considered. This last case is difficult to know for large groups of species, so the interpretation of the results obtained must be done with caution. The descriptors best related to the invasion success of naturalised species within each region present a very similar pattern in the MCRs.

For California, central Chile and S-W Australia the most important predictor of the species’ invasion success is always minimum residence time, a fact consistent with many other studies^5,10,54,55^. Residence time integrates a number of factors (increasing propagule pressure and allowing enough time to adapt to new conditions, which allows overcoming a lag phase), which enhances the chances of colonising new territories^54–56^. Once the effect of species’ time of residence has been considered, the variable that best predicts intraregional invasion success in these three MCRs is high occurrence in the region of origin. The highest level of intraregional invasion success occurs when the species has been introduced long time ago (more than 100 years) and especially if the occurrence of the species in the Iberian peninsula is very high, which would increase the chances of arrival of the species^9,52^. In the case of California and SW Australia, other geographical variables are added to the time of residence and the occurrence to determine the success of the invasion: a broad latitudinal range (for the naturalised species of California) or from southern regions on the Iberian Peninsula (in the case of Australia). As with inter-regional success, more ecologically versatile species, capable of living under very different environments in the Iberian Peninsula, are also capable of occupying a greater part of the available range in California. In contrast, the naturalised species exhibiting the highest degree of success in S-W Australia are not the most ecologically versatile, but those associated preferentially with the south of the Iberian Peninsula (i.e. hot-summer Mediterranean climate), a fact that might be associated with certain climatic particularities in Australia. In S-W Australia the absence of cold marine currents and the moderate relief prevent frost in winter and make the summer hotter^57^ which could favour species coming from warmer Iberian territories. Additionally, unlike the rest of the MCRs (except South Africa) S-W Australia has no continental masses at higher latitude (towards the pole) and therefore no oceanic climatic territories that could house species with higher precipitation needs, such as the characteristic species of the northern Iberian Peninsula.

The above analyses provide a particular insight into the drivers (patterns and processes) of herbaceous plant invasion success among the five MCRs.

The characterization of the exotic species in their region of origin may shed light on how the invasion processes will occur: which species are potentially more successful in the colonization/naturalization of other territories as well as their potential to spread into these new areas. All this knowledge is essential in decision making to anticipate and handle possible scenarios of future plant invasions.

## Methods

### Flora of the Mediterranean regions

Mediterranean-climate regions (MCRs) are characterized by a unique climate, with wet, cool winters and long warm, dry summers^58^. In order to demarcate the five MCRs we considered administrative boundaries as it is to these that many of the available data refer (Supplementary Note 2). As a representative of the Mediterranean Basin, we chose the Iberian Peninsula because of its historical relationship with California and Central Chile (Supplementary Note 1) and because it has an area of the same order of magnitude as California, S-W Australia and, to a lesser extent, Central Chile (Supplementary Table 2).

Within the Iberian Peninsula we considered peninsular Spain, the Balearic Isles and Portugal, excluding the north provinces which have an exclusively oceanic climate. For California we contemplated the state boundaries. In Chile we selected the seven central regions (from Atacama region to Bio-Bio region) presenting a Mediterranean climate. For South Africa we considered the Western Cape province. Finally, in Australia we selected both the Mediterranean part of the state of Western Australia and that of the state of South Australia. Although the exotic species lists from both Australian regions are relatively different (55% of species in common) and the dates of entry into each of the two regions also differ (on average 20 years earlier in South Australia than in Western Australia), we preferred to combine both sub-regions in a single MCR.

From each MCR we drew up a list of exotic species (Supplementary Note 3). We only considered the naturalised exotic species, i.e., those capable of maintaining viable populations without human intervention^59^. Furthermore, we only considered species with records for at least the last 40 years (Supplementary Note 3). For the Iberian Peninsula we included only the neophytes, that is to say, exotic plants introduced after the year 1500. We did not consider hybrids, and in the case of sub-species or varieties, we assigned the name accepted at species level. We only considered herbaceous species because of constituting the dominant life form among the exotic plants within the Mediterranean Biome^30^ and due to the fact that invasion pathway and success can be highly influenced by life form^36^. We did not consider ferns. We did not consider aquatic plants either, as these present highly specific ecological requirements. Using all the above-mentioned criteria, we attempted to control for some of the factors that might influence the naturalisation capacity, and therefore the invasion success of the species.

For each MCR, each species was also assigned a minimum residence time, considered to be the difference between 2017 and the year in which it was first cited in that MCR. Using the first citation to estimate the time of arrival of a species could involve bias for different reasons (among others, time lag between the date of introduction and collection, accessibility of field sites, variability of sampling efforts over time^60^), although in our case this does not appear to have differentially influenced the naturalised species in relation to the native ones (see Supplementary Note 4). We also assigned a possible pathway category. The Convention on Biological Diversity proposes six principal pathway categories: release, escape, contaminant, stowaway, corridor and unaided^61^. Nonetheless, in most cases it is difficult to establish the specific manner of entry into each MCR^39^. In addition, the information provided by different sources, such as the World Economic Plants database^62^ might not necessarily be applicable to a given region. For this reason we chose to differentiate three situations that are more reliable and easier to assign: species introduced for ornamental purposes, those employed as crops, and the remaining unintentionally introduced species, regardless of their pathway of arrival. For the ornamental and crop species, which would constitute particular cases of escape or release respectively, we only considered those that were clearly introduced with this intention (e.g. with a commercial interest), otherwise, they were assigned to the “unintentional” category. This classification of introduction pathway was used to display the degree of naturalisation of species in each MCR (Table 1); for the rest of the analyses only unintentionally introduced species were considered.

### Characterisation of Iberian native species

The results of this study highlight the Iberian Peninsula as a main donor of exotic species to the other four MCRs, and analysis of invasion success was therefore conducted only for species native to this region. To this end we employed data from 12,573 relevés obtained by means of extensive bibliographic revision and from the SIVIM (Iberian and Macaronesian Vegetation Information System; http://www.sivim.info/sivi/). The relevés were widely distributed through each province in both countries that are included in the Iberia Peninsula^63^. Only relevés performed in communities dominated by herbaceous plants were considered. For more information on this dataset see Casado *et al*.^63^. This dataset contains 2,664 native Iberian herbaceous species, 681 of which are present as naturalised species in at least one of the other four MCRs.

Each of the 2,664 species were characterised on the basis of attributes widely recognised for their importance in the invasiveness of the species^8–11^. The selection was made taking into account the information available for this large set of species. Thus, we assigned the life cycle (annual, biennial or perennial), according to the information available for the different regional floras (See Supplementary Note 3). We also assigned different variables related to their spatial distribution at different scales. At local scale we calculated cover (average and maximum, in percentages) that the species presented in the relevés where it was present. This cover value represents the species’ capacity to become dominant at the plot scale. At a broader scale we noted the number of relevés in which the species was present, which provides information, among others, on the chance of the species coming into contact with humans, a fact that represents the vector of introduction into the recipient area^9^. Likewise, we calculated the latitudinal (average and range, in km) and altitudinal (average and range, in m) distributions on the Iberian Peninsula, considering all the relevés in which the species was present. Latitudinal and altitudinal distributions provide information on a species’ versatility in relation to environmental variability. Moreover, we only calculated the values for range when the species was present in at least 10 relevés, which corresponds to 86% of the species present as naturalised in other MCRs. For the remaining 14% of the species the values of range were recorded as missing data. Finally, at the global scale, we created an ordinal variable with six categories which represented concentric belts according to the extent of the species’ biogeographical distribution: (1) endemic to the Iberian Peninsula; (2) present in Iberia and southern France or North Africa; (3) W of the Mediterranean (as far as Italy); (4) belonging to the Mediterranean Basin, as far as the Middle East; (5) Eurasian and (6) cosmopolitan distribution. See Supplementary Note 3 to consult the sources from which the data on the distribution of the species in the region of origin have been extracted.

### Data analysis

We analysed the species flows among the different regions considering the species native to a given MCR and naturalised in any of the other four MCRs. The value observed for each flow was compared with the values expected under a hypothesis of randomness. Following van Kleunen *et al*.^1^ we created a pool with all the naturalised species from the five MCRs together, indicating for each one the region in which it is native: Iberian Peninsula, California, Central Chile, Western Cape, S-W Australia or another region. In order to assess the flows, for each MCR we eliminated its native species from the pool and randomly selected a number of species equal to that of the naturalised ones in that MCR, recording for each of them the region of origin. This process was repeated 999 times for each MCR. We differentiated flows observed to be greater or lower than the randomly expected ones when they were greater than the 97.5th percentile or lower than the 2.5th percentile, respectively.

In a second step we analysed the factors determining the species’ invasion success. Given that the Iberian Peninsula constitutes the principal donor of species to the remaining MCRs, these analyses have been focused on this region. As observations we employed the 2,664 herbaceous species native to Iberia that were present in the 12,573 relevés. We used two response variables: invasion success calculated at both interregional and intraregional scale. Interregional success was evaluated as the number of MCRs in which this species appeared as naturalised: from a value of 0 when it was only present in Iberia as native to 4 when it was present as naturalised in the other four MCRs. As descriptors we used life cycle, the geographical distribution on the Iberian Peninsula obtained from the relevés (number of relevés in which the species was present, average and maximum cover, average latitude and range, average altitude and range) and the biogeographic distribution belts (above mentioned ordinal variable of six categories). Intraregional success was calculated for each MCR as a measure reflecting potential regional spread, considering the percentage of territorial units where the species were present. This percentage was calculated for the 58 counties of California, Chile’s seven central regions, eight botanical regions of South Australia and seven IBRAs of Western Australia (see Supplementary Fig. 2 and Note 2). In the case of Western Cape, intraregional success could not be calculated due to the impossibility of assigning territorial units to exotic species within this MCR because of the lack of data available at spatial scales smaller than the country’s provinces and because the herbaceous taxa are underrepresented in relation to the woody species^54^. Thus, attempting to calculate intraregional success for this region would have involved considerable less accuracy and much greater geographical environmental variability than in the remaining MCRs. For the intraregional success analyses we employed the same descriptors than in the analysis of interregional success, but we also added minimum residence time.

In both cases we used Classification and Regression Trees (CART) to predict species’ degree of invasion success according to the different predictors^64^. Given the unbalanced data existing between the 5 categories of the interregional invasion success this response variable was weighted taking into account the number of species in each category. CART was widely used as a tool for predicting invasiveness based upon a set of predictor^20,40,55^. CART constitutes a non-parametric technique that enables us to hierarchize the combination of predictors that best discriminates the response variable. It is not influenced by outliers or missing data and it can detect complex interactions among continuous and categorical descriptors^65^. This analysis considers each predictor separately selecting the one with the greatest discriminatory capacity. Once a certain predictor has been chosen, the others will not be chosen unless they provide non-redundant information. For this reasons multi-collinearity between independent variables is assumed to be handled automatically by the nature of CART^66,67^. In order to indicate the fit of the model, risk estimated value has been used. Risk estimate measures the impurity of the tree which is given by the sum of the impurity measures of all terminal nodes. In the case of regression trees, the proportion of explained variance can be calculated by subtracting the risk estimate from one^68^^.^

We performed regression trees with SPSS 22 and used Chaid as the growing method, cross validation, a minimum number of cases in each subsidiary node of 10, as well as a minimum improvement value of 0.01.

## Electronic supplementary material

Supplementary Information

## Data Availability

Data used in the analyses are provided in the accompanying source data file.
