# Supplementary material for: Asymmetric flows and drivers of herbaceous plant invasion success among Mediterranean-climate regions
Source: Sci Rep. 2018 Nov 15;8:16834. doi: 10.1038/s41598-018-35294-7 (PMC6237776; doi:10.1038/s41598-018-35294-7)
Supplement: Supplementary file 1 — Supplementary Information [file 41598_2018_35294_MOESM1_ESM.pdf]

Supplementary Information

Asymmetric flows and drivers of herbaceous plant invasion success among Mediterranean-climate regions

Miguel A. Casado[1*], Irene Martín-Forés[1,2], Isabel Castro[3], José M. de Miguel[1] & Belén Acosta-Gallo[1]

[1] Ecology, Department of Biodiversity, Ecology and Evolution, Complutense University of Madrid, 28040 Madrid, Spain.

[2] Department of Biogeography and Global Change, National Museum of Natural Sciences, Spanish National Research Council, 28006 Madrid, Spain.[3] Department of Ecology, Autonomous University of Madrid, 28049 Madrid, Spain.

*__Corresponding author Address:__
Dr. M. A. Casado,
Department of Biodiversity, Ecology and Evolution,
Complutense University of Madrid,
28040 Madrid,
Spain.
*Corresponding author E-mail: mcasado@ucm.es (M.A. Casado)

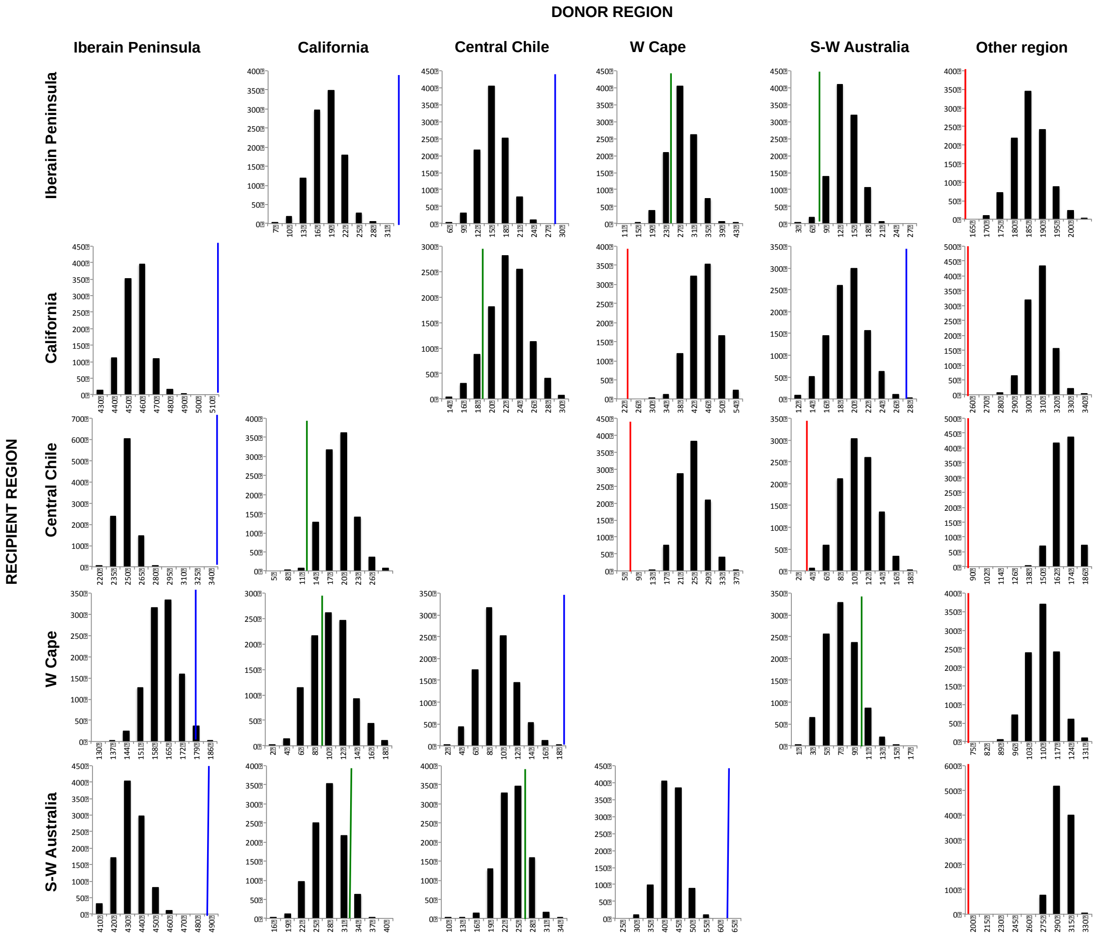

Supplementary Figure 1. Values observed and expected of the number of species naturalised in each of the recipient Mediterranean climate regions (MCRs) coming from each donor MCR. We created a pool with all the naturalised species from the five MCRs, indicating the region of origin for each one: Iberian Peninsula, California, Central Chile, Western Cape, S-W Australia or another region. The value observed for the flow of species between two MCRs was compared with the expected values under a hypothesis of randomness. For the randomness analysis we removed from the general pool of naturalised species those that were native in a given MCR, and then randomly selecting a number equal to that of naturalised species of that MCR, recording their region of origin. We differentiated flows observed to be statistically greater or lower than those expected at random when they were greater than the 97.5th percentile or lower than the 2.5th percentile, respectively. The black bars of the histogram indicate the frequency of values observed based on 999 random simulations. The observed value is shown as a blue vertical line (significantly greater than the expected value), a red vertical line (significantly lower than the expected value) or a green vertical line (value observed within the 95% range of the expected values).

Supplementary Table 1. List of the 28 species native to the Iberian Peninsula with greater invasiveness at interregional scale but not present in any of the other four Mediterranean regions. Greater invasion success corresponds to species with greater latitudinal range in the Iberian Peninsula, a higher occurrence frequency in the relevés and a Eurasian or cosmopolitan geographic distribution. For each species the number of herbarium records outside the respective Mediterranean region of each country is shown, except in the case of Chile, for which the number of regions where it is present is shown.

Species§	USA	South Africa	Australia	Chile
Achillea odorata L.	0	0	0	0
Acinos alpinus Moench.	0	0	1	0
Allium sphaerocephalon L.	4	0	1	0
Anthyllis vulneraria L.	27	0	51	0
Deschampsia flexuosa (L.) Trin.	496	9	2	3*
Campanula rapunculus L.	3	0	0	0
Carex flacca Schreb.	15	0	48	0
Carum verticillatum (L.) W.D.J.Koch	0	0	0	0
Clinopodium vulgare L.	565	0	7	0
Epilobium hirsutum L.	214	264*	25	0
Hornungia petraea (L.) Rchb.	0	0	0	0
Jasione montana L.	0	0	0	0
Juncus inflexus L.	23	60*	13	0
Juncus tenageia Ehrh. ex L.f.	0	0	0	0
Lactuca viminea (L.) J.Presl & C.Presl	0	0	0	0
Lycopus europaeus L.	36	0	4	1***
Minuartia hybrida (Vill.) Schischk.	0	0	0	0
Molinia caerulea (L.) Moench	66	0	4	0
Myosotis ramosissima Rochel	0	0	2	1**
Ononis spinosa L.	0	0	0	0
Prunella laciniata (L.) L.	8	0	18	0
Pulicaria dysenterica (L.) Gaertn.	1	0	0	0
Radiola linoides Roth	1	0	0	0
Ranunculus bulbosus L.	559	0	0	0
Saxifraga granulata L.	1	0	0	0
Saxifraga tridactylites L.	2	0	0	0
Scirpoides holoschoenus (L.) Soják	2	51	0	0
Stachys officinalis (L.) Trevis.	8	0	0	0

§. Data from the GBIF (http://www.gbif.org/occurrence/) for the USA, Australia and South Africa and from Flora del Cono Sur (http://www.darwin.edu.ar/Proyectos/FloraArgentina/fa.htm) and the Laboratorio de Invasiones Biológicas (http://www.lib.udec.cl/search/index.php) for Chile.
* Native in the country
** Only in the Magallanes region
*** Only in the Los Lagos region

Supplementary Table 2. Characterisation of the five Mediterranean regions. For each one, the area considered to create the list of naturalised species is shown. The administrative units correspond to the division of each study area into smaller units in order to calculate colonisation success (see Supplementary Fig. 2).

Region	Area (km²)	Latitudinal range	Administrative units	Population
Iberian Peninsula	544,860	36º to 43º N	Two countries: 53 provinces	49,887,297
California	423,970	32º to 42º N	The state: 58 counties	39,250,017
Central Chile	231,307	25º to 39º 'S	Central Chile: 7 regions	14,446,467
W Cape	129,462	30º to 35º S	No data available	5,822,734
S Australia	232,694	30º to 38 ºS	Part of the state: 8 botanical regions	1,320,000
W Australia	319,343	25º to 35º S	Southwest province: 7 IBRA	2,404,604

Supplementary Note 1. Relevant historical events for an understanding of the exchange of species between the five MCRs

The history of human activity in each MCR is of great importance in the movement and establishment of exotic species. On one hand, modification of the ecosystems for agriculture and livestock farming conditions the invisibility of the territory. On the other hand, movement of products favours the deliberate or unintentional mobilisation of species. We now provide a summary of the principal historical events exerting the greatest influence on disturbance of the territory and on species movement. Special attention is given to the history of European colonisation outside the Mediterranean Basin, given its influence on the movement of species among the different MCRs of the planet.

Iberian Peninsula. In the context of the arrival of exotic species, this process likely began in the Neolithic. Since the origins of agriculture in the Middle East around 10,500 years ago, the associated culture, technology, domestic species (wheat, barley, legumes, cows, goats, sheep and pigs) and flora gradually spread westwards[69-70], reaching the Iberian Peninsula 6,800 years ago[71]. This spread of agriculture cohabited with, and gradually replaced, the hunter-gatherer societies. The use of land for pastures and crops became widespread during Roman times, farming becoming organised around villas that promoted cereal crops and vines. Nonetheless, 2,000 years ago the degree of alteration in the territory was not very high, given Iberia's low population density. Indeed, during the times of dependence upon Rome and following the fall of the Roman Empire (3rd century) and the Visigoth invasions of Central Europe (5th century) there was even a period of regression of agriculture. This tendency prevailed until the 16th century, with alternating periods of expansion and standstill[72]. Thus, the Muslim conquest of the Iberian Peninsula in the year 711 gave rise to increased agricultural activity, promoting, apart from wheat, other species such as olive and orange trees. Likewise, some phases of the Reconquest, like for example from the years 900 to 1300, involved southward migrations of the population which in turn led to greater food requirements. Following the discovery of America in 1492 Spain and Portugal initiated a phase of colonisation on a worldwide scale that culminated in the 18th century and in which they came to dominate over 10% of the total surface area of all the continents. This had two important consequences: 1) connection between species and all the regions of the world through the port of Seville and subsequently of Cadiz and 2) an unprecedented deforestation process on the Iberian Peninsula to supply the merchant

marine and the navy with timber. More recently, in the 19th century, the successive desamortizations (among others, those of Godoy in 1798, of Mendizábal in 1836 and particularly of Madoz in 1855) involved an increase in the area used for cultivation, whilst the industrial revolution (as from the 1830s) provided more advanced technology for ploughing land that had theretofore been unavailable to agriculture. Both these situations led to intense deforestation and unprecedented changes in land uses.

California. Although Hernán Cortés had already visited the California Peninsula in 1532, it was not until 1542 that Juan Rodríguez Cabrillo explored the Californian coast, discovering a ‘very good safe port’, San Diego Bay. Nonetheless, the region was not colonised until 1769 when Fray Junípero Serra founded the first Franciscan mission in the San Diego area. Spain continued to dominate until 1821, when the new state of Mexico was created. In 1846 a group of Americans under the orders of Richard H. Dana and John Sutter, as well as of colonel Mariano Vallejo, declared the independent Republic of California. At that time there were very few inhabitants of European origin, possibly around 4,000, although vast expanses of the territory were occupied by large herds of cattle and flocks of sheep[73]. Shortly afterwards, in 1848, gold was discovered and in just a few years the population rose sharply due to the arrival of approximately 90,000 American immigrants. With this population increase came increased demand for food crops, some of which were supplied by other countries, such as Chile[45], at least until the transcontinental railway was built in North America and the Panama Canal was opened up[46]. By 1850 California had been officially declared a state of the USA and 1865 saw the start of the state’s industrial development. During the 1850s the population rose from 100,000 inhabitants to 400,000, and by 1890 it had risen to over one million. These changes also affected land uses, and in the 1880s extensive livestock farming gave way to agriculture and housing urbanisation, a trend that continues to the present day.

Australia. The first European sighting of the continent was possibly by the Dutch sailor Willem Janszoon in 1606. However, although in the next 160 years there were several European expeditions, it was not until 1770 that the Endeavour expedition led by James Cook first disembarked on the continent in Botany Bay. Cook then headed northwards, disembarking on Possession Island, where he officially claimed Australia’s east coast for Great Britain under the name New South Wales. The first settlement and penal colony were set up in Port Jackson (modern-day Sidney) in 1788. The western and southern states of Australia were founded from the start as free states: firstly, the

west of Australia, claimed in 1828 by the United Kingdom under the name Swan River Colony, in 1832 becoming Western Australia, and subsequently, in 1836 South Australia, split off from the territory of New South Wales. In the case of W Australia the first settlement was set up in 1827 at King George's Sound (currently Albany), and still belongs to New South Wales. Shortly afterwards, and as independent colonies, Fremantle and Perth were established, with little more than 1,500 inhabitants in 1832. The population grew slowly until in the 1890s the discovery of gold in Coolgardie caused another influx of immigrants. South Australia was created by means of an Act approved by the British parliament in 1834 permitting the establishment of the province of South Australia; it had an area of 802,511 km^2 and was free of convicts. The new province was established in Kingscote (Kangaroo Island) in July 1836, although five months later, the site of present-day Adelaide was chosen as the official settlement of the colony. The first immigrants reached the bay of Holdfast at the end of 1836 and several days later the colony was proclaimed.

Western Cape. Europeans first came into contact with the region of the Cape of Good Hope in 1487 on the Portuguese expedition of Bartolomé Díaz and 10 years after, during the first voyage to India of the navigator Vasco de Gama. However, the region saw little contact with Europeans until 50 years later, when in 1652 the Dutchman Jan van Riebeeck and other employees of the Dutch East India Company were sent to the Cape to set up a supply station for the route taken by their ships to the Dutch East Indies. This settlement led to a change in the way natural resources were exploited, from subsistence nomad pastoralism to sedentarisation, with crops and agricultural techniques imported from Europe[74]. However, as the region presented very poor soils, vegetables and cereal crops were not widely grown, which led to a slow colonisation process. Nonetheless, this small Dutch trading colony gradually grew over the next two centuries, and had a population of 26,000 of European origin by the end of the 18th century. The British invaded the region in 1797 during the fourth Anglo-Dutch war. Having been returned to Holland in 1803 it was once again occupied by Great Britain in 1806, becoming a British colony from 1814 to 1910, when it was created and incorporated into the South African Union. During this time as a British colony, diamond (1867) and gold (1886) mines were discovered, giving rise to strong economic growth and mass immigration.

Chile. Fernando de Magallanes and his 1520 expedition were the first Europeans to reach Chile from the south through the straits that now bear his name. Shortly

afterwards, in 1536, Diego de Almagro led an overland expedition from the north to the Aconcagua Valley and the north of present-day Chile. In 1540, Pedro de Valdivia, authorised by Francisco Pizarro, conducted a second expedition, this time through the Atacama desert, which represented the start of the Conquest. On reaching the Copiapó valley, he took solemn possession of this land in the name of the King of Spain, calling it Nueva Extremadura, in memory of his homeland. He then marched towards the Aconcagua valley, and on February 12th 1541, established the basis of the city of Santiago del Nuevo Extremo, the modern-day Santiago de Chile. In the next 60 years the conquest of the country continued, with the setting up of the main cities: Valparaíso (1542), La Serena (1544), Concepción (1550), Valdivia (1552), Villarrica (1552), Los Confines (1553) or San Bartolomé de Gamboa, currently known as Chillán (1580). Lands were gradually conquered southwards as far as the banks of the Bío-Bío river, where war was waged against the Mapuche people; this border was maintained until 1818. In 1600 the period known as the Conquest came to an end and the Spanish colonial period began, lasting until the country's independence in 1810. Since the colony was established agriculture was promoted (mainly wheat crops and extensive livestock farming), this became more intense in time depending upon population density and on demand from international markets. Thus, two specific periods led to the intensification of agriculture. Firstly, at the end of the 17th century the Viceroy of Peru requested all the wheat Chile could provide, with exports reaching 17 t/year by the end of 1680s[75]. Subsequently, at the end of the 19th century, the country now having gained its independence and with the 1848 California gold rush in progress[76], in 1880 Australia once again promoted the intensification of agriculture, becoming one of the three principal wheat exporters[77]. Following these events, agricultural development stagnated in Chile until the advent of modern agriculture halfway through the 20th century[78] and the mechanisation of the countryside as from 1980[79].

Supplementary Note 2: Study area

For each of the five MCRs we delimited spatial units to quantify colonisation success considering the limitations of the available data. For the Iberian Peninsula we selected the Mediterranean-climate area corresponding to two countries, Spain and Portugal[80], excluding the provinces with an exclusively oceanic climate (A Coruña, Lugo, Asturias, Cantabria, Bizkaia and Gipuzkoa). Therefore, we used as spatial units the 42 provinces of Spain and the 11 provinces of Portugal (Supplementary Fig. 2). In North America we considered the California Floristic Province which, excluding its area of southern Oregon and northwestern Baja California, corresponds approximately to the political boundaries of the State of California[31]. Within this State we considered the 58 counties as spatial units, although not all of them included this Floristic Province within their territory. As for Chile, the area selected corresponded to the seven central regions presenting, at least partially, a Mediterranean climate: Atacama, Coquimbo, Valparaíso, O'Higgins, Maule, Biobío and the Santiago Metropolitan Zone. In the case of South Africa the study area corresponded to the province of Western Cape which includes most of the Cape Floristic Region. In South Africa it was not possible to obtain spatial units presenting greater spatial detail than the province, therefore Western Cape could not be included for intraregional success analyses. Finally, in Australia we considered the Mediterranean areas, both of the state of South Australia and of Western Australia. For South Australia, given the information available, we considered the botanical regions of South Australia[81] selecting the eight regions exhibiting a Mediterranean climate: Flinders Ranges, Eyre peninsula, Northern Lofty, Murray, Yorke peninsula, Southern Lofty, Kangaroo Island and South-Eastern. For Western Australia we selected the South-West province and within this, the seven IBRAs (Interim Biogeographic Regionalisation of Australia[82]) constituted the spatial units regions: Avon Wheatbelt, Esperance Plains, Geraldton Sand plains, Jarrah Forest, Mallee, Swan Coastal Plain and Warren.

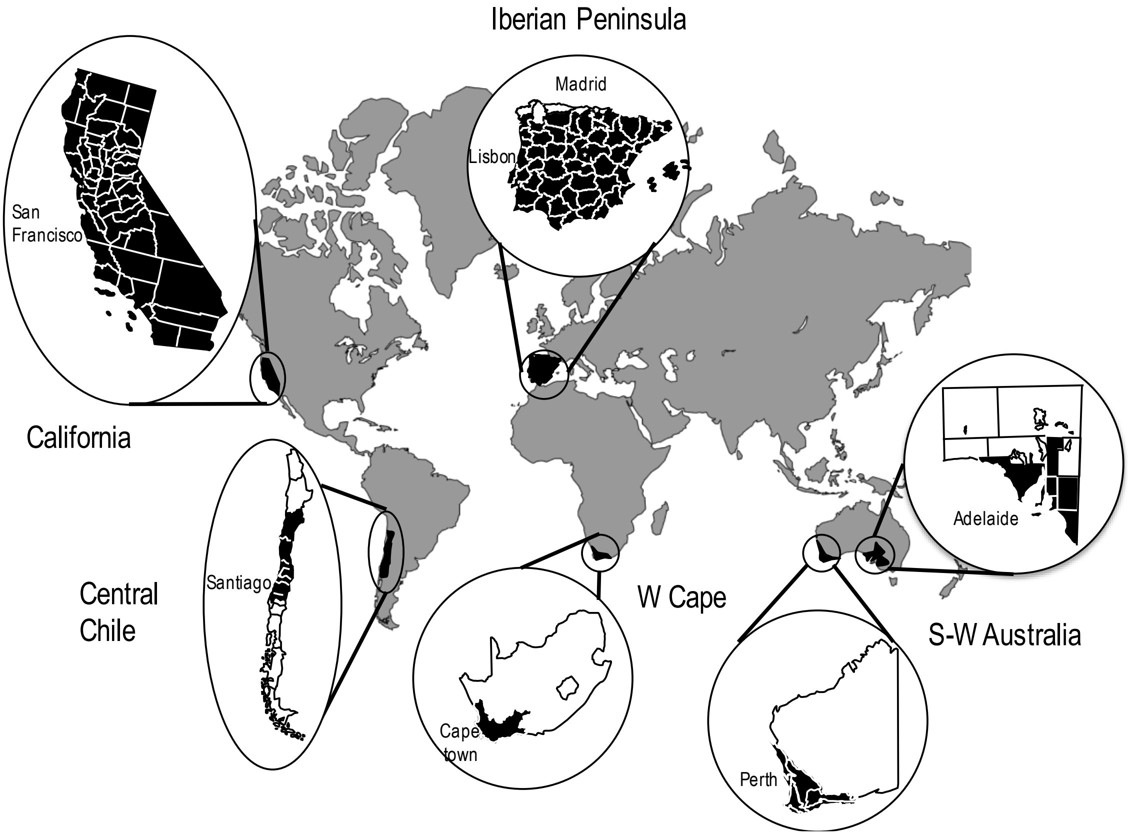

Supplementary Figure 2. Boundaries of the five regions considered in the present study. In all cases the area in black indicates the area considered to create the list of naturalised species. The divisions within each region indicate the territorial units used to calculate each species' colonisation success. For California these are the counties, for the Iberian Peninsula the provinces, for Central Chile the regions and for Australia, the IBRA or botanical regions. In the cases of Western Cape, intraregional success was not evaluated due to the fact that it was not possible to obtain spatial units presenting greater spatial detail than the province.

Supplementary Note 3: data on the naturalised species

The data on the presence of naturalised species and their distribution within each MCR were extracted from the following sources:

1) Iberian Peninsula: We used the information from the Atlas on Spain's invasive allochthonous plants by Sanz Elorza et al.[83], complementing and updating it with Flora Iberica (http://www.floraiberica.es/) and the System of information on the plants of Spain, Anthos (http://www.anthos.es/). The number of provinces in which it was present was extracted from Flora Ibérica, and for the taxa not yet published in the present research, from Anthos.

2) California: We employed "Calflora" (https://www.calflora.org/), selecting as our search criteria "Not native to California" and "Grasslike, Herb, Vine". We also extracted from Calflora the number of counties in which each species was cited. The citations include both data on herbarium collections and personal observations.

3) Central Chile: Our principal source of data was the catalogue of Chilean flora by Marticorena & Quezada[84] and the Database on invasive species in Chile from the LIB (*Laboratorio de Invasiones Biológicas;* http://www.lib.udec.cl/search/index.php) of the University of Concepción[85]. Both for the list of species and for their distribution in the different regions of the country, the information was extracted from the LIB and from the works of Castro et al.[5] and Fuentes et al.[85]; these were complemented with the data provided by the Catalogue of vascular plants of the Southern Cone, of the Darwinian Botanical Institute[86].

4) Western Cape: We extracted the list of naturalised species from "Plants of Southern Africa, an online checklist" (http://posa.sanbi.org/intro_precis.php), selecting "Life form" (herb / climber), and Western Cape as the region. Of the full list of species present in this region we selected only the ones that are naturalised in this province.

5) Australia: For South Australia we used the "Electronic Flora of South Australia" (http://www.flora.sa.gov.au/census.shtml), selecting the option "naturalised". We then selected only the herbaceous species. We only considered the species present in at least one of the 8 botanical regions selected for this state. For Western Australia we used "Florabase: the western Australian flora" (https://florabase.dpaw.wa.gov.au/search/advanced), selecting the criteria "Alien" (yes) and "Habit" (climber / herb). We only considered the species present in the South-West province, recording the IBRA regions in which they were present.

For each species we searched for the region of origin by consulting different regional floras, including Flora Iberica[87], Flora Europaea[88], Flora del Cono Sur[86], as well as information from different databases, mainly eMonocot (http://emonocot.org), the U.S. National Plant Germplasm System (https://npgsweb.ars-grin.gov/gringlobal/taxon/taxonomysearch.aspx) and the Kew World Checklist (http://apps.kew.org/wcsp/advanced.do).

For each species we found the dates of the first and last record in each MCR, obtained from herbarium collections and other data collections[89]. As first record we considered the oldest herbarium specimen, except if between this and the date of the following one, over 100 years had transpired. These cases can correspond to failed introductions or initial observations in botanical gardens which had not actually become naturalised, and we therefore excluded them from the study. We used first record date to estimate Minimum Residence Time[90] as the difference in years between 2017 and the first record date. This estimate is underestimated, given that the species was definitely present prior to its first collection. We used first record date to estimate the presence of the species at the present time.

For the study we only considered species cited in at least the last 40 years (after 1976). Although this cut-off for considering the species to be naturalised is arbitrary, we believe it is appropriate since it allows enough number of generations for species that are predominantly annuals and it is of the same order of magnitude than the lag phase in invasive species[5,22]. For the Iberian Peninsula and Western Cape we took the record data from the Global Biodiversity Information Facility (GBIF) (http://www.gbif.org/occurrence/search), seeking the occurrences of each species and applying the filters "specimen" and "country" (Spain + Portugal or South Africa, respectively). For California we employed the database of the Consortium of California Herbaria (http://ucjeps.berkeley.edu/consortium/). In the case of the two Australian regions we used Australia's Virtual Herbarium (http://avh.chah.org.au/), noting separately the records corresponding to South Australia and Western Australia. In both cases the dates considered correspond to citations for the whole state, not for the IBRA and the botanical regions considered. For both the consultations of the GBIF and of the AVH and the Consortium of California Herbaria, we performed a purge of potential errors in the databases, contrasting the data on collection dates with the record collectors. Thus we detected and eliminated numerous errors, particularly in the older citations. Finally, in the case of Chile, we only availed of the information from the first

record through the LIB, and consequently, the criteria referring to the presence of the species in the last 40 years, or the time elapsed between the first and second citations could not be applied to this region. In relation to the remaining regions, this overestimates the number of naturalised species present.

With regard to nomenclature, the five species lists obtained were standardised. Hybrids were excluded. We only considered the taxonomic level of the species, and subspecies and/or varieties were assigned to the corresponding species. In order to avoid synonymous names among species of different origin, we conducted an exhaustive search for equivalences among names. Given that most of the species found were present on the Iberian Peninsula, species names were standardised in accordance with the taxonomy of Flora Iberica[87] and for as yet unpublished taxa, of Anthos. In a second step, the species not found in these two databases were sought in the Euro+Med PlantBase (http://www.emplantbase.org/), thus confirming their status as different species. Finally, all species were compared with The Plant List (http://www.theplantlist.org/). The latter step involved the use of the 'Taxon Stand' R package[91].

Supplementary Note 4: temporal sequence of prospection for native and naturalised species in the Mediterranean regions

Using the date of the first record as a species arrival date can involve bias for several reasons. On one hand, herbarium records are usually subsequent to the date of naturalisation of the species in the region. Furthermore, there can be bias associated with identification ambiguity, accessibility of field sites, and variability of sampling efforts over time[59]. This bias can also be seen in a differential manner for native or naturalised species. For instance, in recently colonised and prospected territories, like some of the studied ones, and in particular in California and Australia (see Supplementary Note 1), there can be a time lag between the entry of new species and the scientific activity indicating their presence. That is to say, the initial citation of an naturalised species might not be reflecting its entry into the country, but rather the start of botanical collection. Moreover, botanical activity in a colonised region may very well be biased in favour of prospecting for native species over naturalised ones[92].

For all these reasons, in our study we considered two analyses intended to control differences in botanical collection in these regions. We describe the frequency of plant record collection over time, particularly at the time of the first description of the species in each MCR. To this end we compiled all the plant records up to the year 1870, classifying them according to the year they were collected. Furthermore, analysis of the variability in the sampling effort over time enables us to compare native species with naturalised ones. Our underlying hypothesis states that if the collection activity does not depend upon the type of species, then there should be a time lag between the initial citations of native and naturalised species. To verify this hypothesis we recorded the date of the first citation of all the unintentionally introduced species and for the native species we randomly selected a subset of 150 species within a pool comprising native species selected by means of the same criteria as the naturalised ones: herbaceous plants, non aquatic ones, non hybrids or levels below that of species. To this subset of species we assigned the date of the first record according to the criteria employed for naturalised species. For Central Chile we had no access to the country's herbaria and we did not therefore avail of data on the native species.

The rate at which records are incorporated until 1870 differs greatly in the different MCRs, although it presents certain patterns in common: in all cases there is an initial time period with very little prospection which only occasionally presents moments of botanical activity (Supplementary Fig. 3). Subsequently, on dates varying

according to MCRs, there has been practically continuous activity that has lasted up to the present time. On the Iberian Peninsula the first citations are from 1600, with five records by the German botanist Joachim Burser. Subsequently there were very few citations until the contributions made by the French botanist Antoine Laurent de Jussieu in 1716-17, the Swede Pehr Osbeck in 1751, the Scotsman Francis Masson in 1776-78 and the Spaniard Diego Lorenzo del Prestamero y Sodupe in 1785. As from 1790, collection is continuous, with a rate of over 150 records/year by renowned botanists like the Frenchman Eugène Bourgeau (4,277 records), the Swiss Pierre Edmond Boissier (954 records) and the Dane Johan Martin Christian Lange (600 records).

In California scientific knowledge in general and botany in particular run parallel to the history of the region (see Supplementary Note 1). The first known citation is from 1764, collected by the Briton William Hudson. Until the end of the 18th century, only occasional collections appeared, associated with the first expeditions during colonial times, such as the French collection of La Pérouse from 1786, the Spanish one of Malaspina from 1791 or the British one of Vancouver of 1792-95. Thus, Tadeo Haenke, of the Malaspina expedition, or Archibald Menzies of the British Vancouver expedition, were the first botanists to collect plants in the region[93], with around ten records each. Botanical research became much more prominent in 1833 with the Briton David Douglas who collected plants for the Horticultural Society of England. He collected 650 specimens in California[94], among these some naturalised plants such as *Bidens pilosa* L. and *Malva parviflora* L. Shortly afterwards and until 1850 several American expeditions were conducted, the most noteworthy of these being that of Captain Charles Wilkes in 1839 and the four conducted by John C. Fremont (1843-1844, 1845-1846, 1848-1849 and 1853-1854). The situation changed when the region was named State of the Union in 1850. Three years later the California Academy of Natural Sciences was founded in San Francisco and in 1868 the University of California was founded. As from 1860 plant collection was continuous and very intense, with a rate of over 1,095 records/year; the most renowned naturalists were Charles C. Parry (223 records), John M. Bigelow (675 records), Henry N. Bolander (4,095 records), William H. Brewer (4,106 records) and Albert Kellogg (1,518 records).

In the case of Australia both regions (Southern and Western) present quite a similar history, although in W Australia botanical prospection was somewhat earlier and more intense in the initial stages. For Western Australia the first specimens collected are those of the Briton William Dampier during his exploration of the Australian coast in

1699, and for South Australia 70 years after, those of Sir Joseph Banks and Daniel Charles Solander in 1770. In 1792 Archibald Menzies, with the British Vancouver expedition, collected around 15 plants on the West Coast of ‘New Holland (King George Sound)’. Subsequently, three naturalists made successive botanical contributions: from 1801 to 1805 the Scotsman Robert Brown, having conducted several botanical expeditions in the region of The Cape, incorporated over 860 records to the flora of both Australian regions; from 1818 to 1822 the Englishman Allan Cunningham conducted multiple expeditions along the Australian coast, collecting approximately 300 records; finally, from 1823 to 1829 William Baxter collected over 180 specimens, especially in the surroundings of King George Sound. As from 1838 a practically continuous record has existed, with a collection rate of approximately 311 records/year. Some of the most renowned naturalists are the German Johann August Ludwig Preiss (2,260 records, among which are naturalised species such as *Chenopodium murale* L. or *Arctotheca calendula* (L.) Levyns), the Scotsman James L. Drummond (920 records) and in particular, the German baron Ferdinand Jacob Heinrich von Mueller (4,682 records, including the naturalised species *Aira praecox* L., *Cynodon dactylon* (L.) Pers. o *Rumex acetosella* L., among many others).

In the region of The Cape the first botanical record is relatively recent, from 1771; it was collected by the Englishman Joseph Banks on the Cape of Good Hope during James Cook’s expedition aboard the HMS Endeavour to the South Seas. Archibald Menzies, a member of George Vancouver’s British expedition, also visited The Cape in 1791, before continuing his voyage to Australia. Shortly afterwards, from 1772 to 1775 the region of the Cape was visited by the Swede Carl Peter Thunberg, a disciple of Carl Linnaeus; this journey led to the publication of his Flora Capensis[95-96]. Subsequently, the Englishman William John Burchell collected around ten records from 1814 to 1815. It was not until 1824 that the German Johann Franz Drège, and especially the Dane Christian Friedrich Ecklon, began to make more significant collections. C. F. Ecklon was a chemist residing in Cape Town who donated 350 herbarium records with which the South African Museum was founded, and which were to constitute the basis of South Africa’s first herbarium[97]. Together with Carl L.P. Zeyher he was responsible for numerous collections. Moreover, J.F. Drège conducted numerous expeditions from 1826 to 1832, citing some already naturalised species such as *Erodium moschatum* (L.) L'Hér. In 1853 Nils Johan Andersson and the American Charles Wright collected

around twenty records each, subsequently maintaining a very low rate of incorporation of 6 records/year up to 1870.

In order to analyse temporal variability in the first citations of native and naturalised species, the first citation dates were grouped according to decades and represented throughout time for each MCR (Supplementary Fig. 4). Although there are citations of both native and naturalised species throughout the whole time period, there tends to be a segregation process in which native species are cited for the first time before the naturalised ones. This is especially notable in the case of the Iberian Peninsula and, to a lesser extent, in California, whereas in S and W Australia, and particularly in Western Cape, there is less segregation of citations between both types of species. Thus, in the four MCRs there are significant differences between the average date of the first citation of native and naturalised species (Supplementary Table 3), with a time lag of between 79 years for the Iberian Peninsula and 20 years for Western Cape. The Iberian Peninsula presents the oldest citations both for native (25th percentile = 1791) and naturalised species (25th percentile = 1847) (Supplementary Table 3), followed by Western Cape, around 30-40 years after. In the case of Central Chile, due to a lack of data on native species, the naturalised ones were first described relatively early, only 7 years later than for the Iberian Peninsula. On the contrary, California and S and W Australia present the most recent dates, around the middle of the 19th century for the 25th percentile for the native species and the end of the 19th century for the naturalised ones. This temporal sequence in time of residence of the naturalised species is very similar to that detected by Visser *et al.*[31] on analysing only species of grasses.

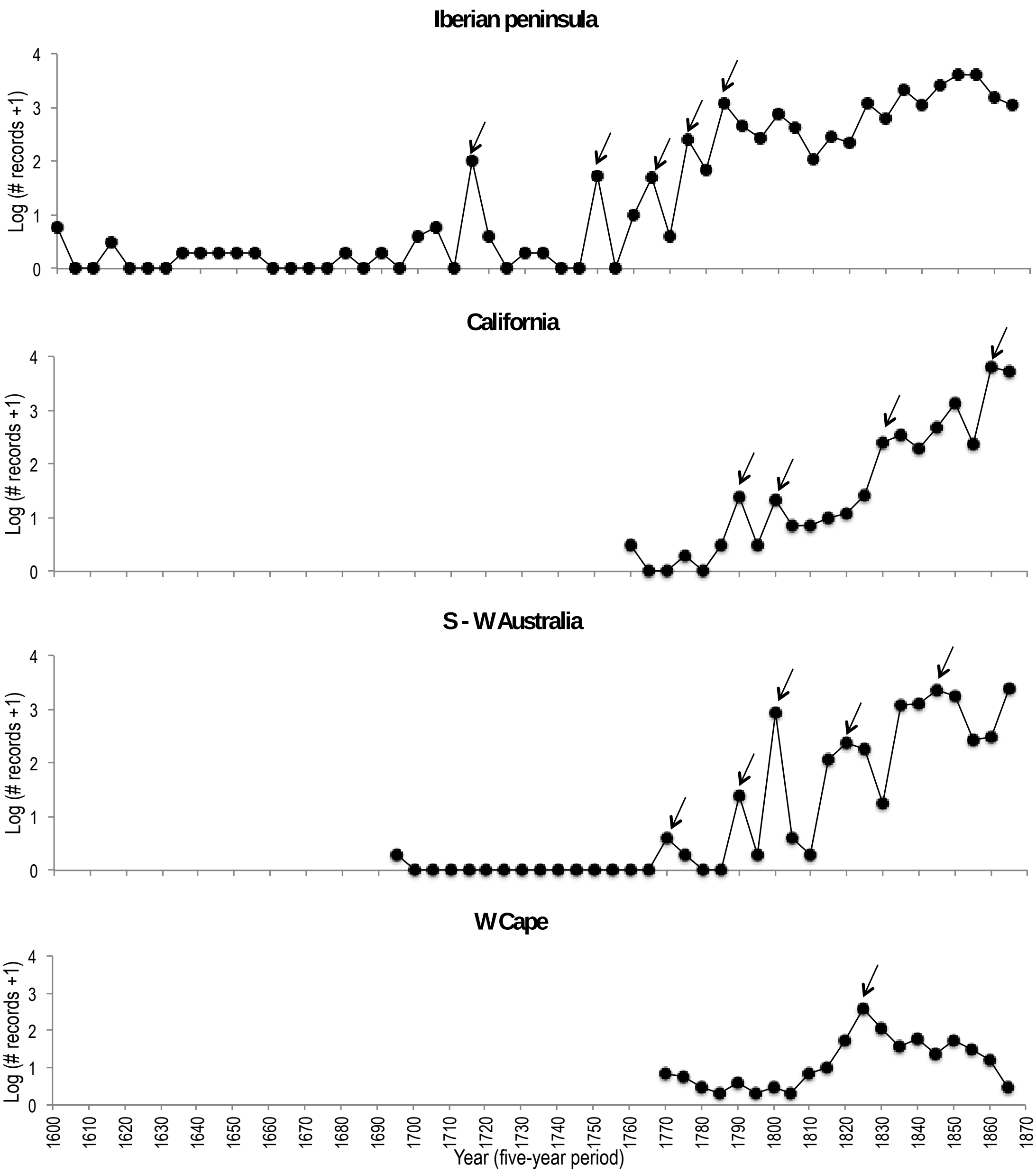

Supplementary Figure 3. Number of plant records (in logarithmic scale) for every 10 years in the different Mediterranean regions. Data are only presented up to 1870. Arrows indicate moments of intense plant collection (see Supplementary Note 4). Data from GBIG (http://www.gbif.org/occurrence/).

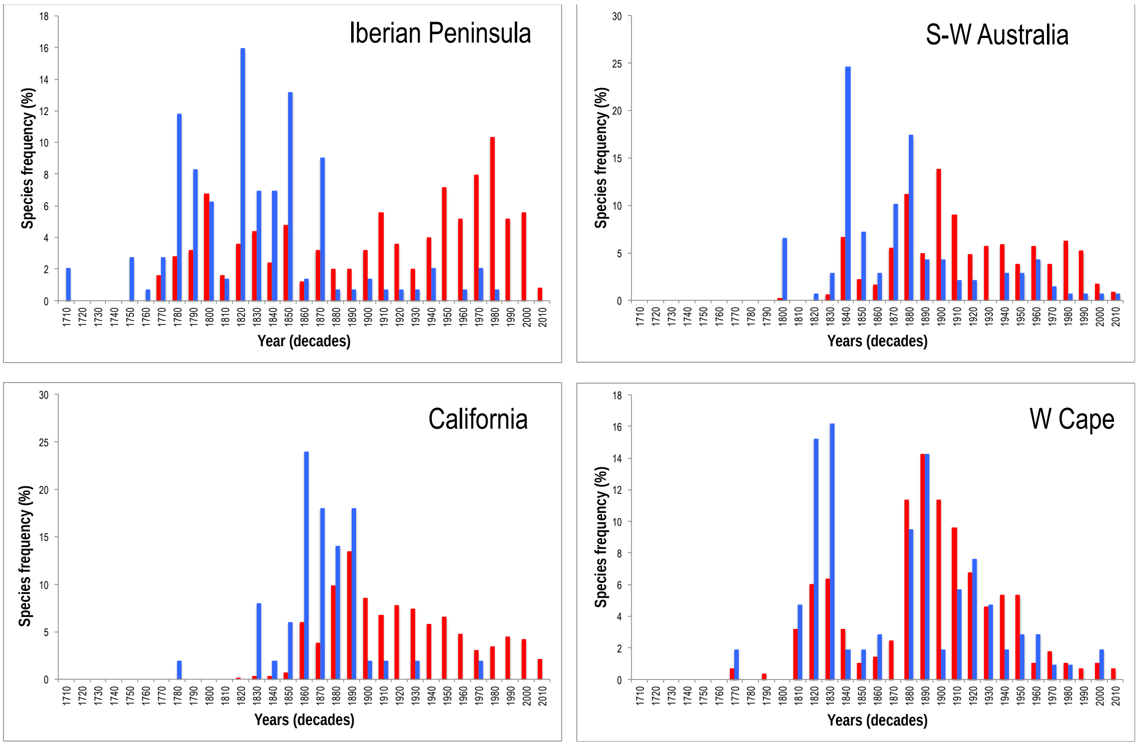

Supplementary Figure 4. Percentage of species described in herbarium records according to the data of the first record. Times are grouped into decades. Native species (blue bars) were differentiated from the naturalised ones (red bars).

Supplementary Table 3. Mean (and standard error) and 25th percentile (P25) of the date of first citation for naturalised and native species in the five Mediterranean regions. The F statistic (ANOVA test) is shown, as well as the associated probability for comparison of the mean values for the two groups of species in each region.

	Native species		Naturalised species			
	Mean (SE)	P25	Mean (SE)	P25	F	p
Iberian Peninsula	1831.8 (4.20)	1791	1910.9 (4.52)	1847	135.03	<0.001
Central Chile	-	-	1907.5 (2.35)	1854	-	-
W Cape	1877.7 (4.99)	1830	1897.3 (2.72)	1879	13.26	<0.001
S and W Australia	1879.3 (3.88)	1848	1920.4 (1.57)	1883	99.56	<0.001
California	1884.0 (3.30)	1863	1924.9 (1.45)	1892	88.24	<0.001

Supplementary references:

69. Sykora, K. V. in *Biological Invasions in Europe and the Mediterranean Basin* (eds di Castri, F. *et al.*) 37-50 (Kluwer Academic Publishers, Dordrecht, 1990).

70. Hancock, J. F. *Plant evolution and the origin of crop species* (CABI, Michigan, 2012).

71. Zilhão, J. The spread of agro-pastoral economies across Mediterranean Europe: A view from the far west. *J. Med. Archaeol.* **6,** 5-63 (1993).

72. Costa, M., Morla, C. & Sain, H. *Los bosques ibéricos. Una interpretación geobotánica* (Planeta, Barcelona, 1997).

73. Rundel, P. W. in *Landscape disturbance and biodiversity in Mediterranean type ecosystems* (eds Rundel, P.W. *et al.*) 3-22 (Springer-Verlag, Berlin, 1998).

74. Davis, G. & Wynberg, R. in *Landscape disturbance and biodiversity in Mediterranean type ecosystems* (eds Rundel, P.W. *et al.*) 65-79 (Springer-Verlag, Berlin, 1998).

75. Bauer, A. J. Expansión económica en una sociedad tradicional: Chile central en el siglo XIX. *Historia (Instituto de Historia, Universidad de Chile)* **9,** 137-235 (1970).

76. Davis, H. California breadstuffs. *The Journal of Political Economy* **2,** 517–535 (1894).

77. Aronson, J. *et al.* in *Landscape disturbance and biodiversity in Mediterranean type ecosystems* (eds Rundel, P.W. *et al.*) 155-168 (Springer-Verlag, Berlin, 1998).

78. Cariola, C. & Sunkel, O. *Un siglo de historia económica de Chile 1830–1930, dos ensayos y una bibliografía* (Ediciones cultura hispánica del Instituto de Cooperación Iberoamericana, Spain, 1982).

79. Fuentes, N., Ugarte, E., Kühn, I. & Klotz, S. Alien plants in Chile: inferring invasion periods from herbarium records. *Biol. Invasions* **10,** 649-657 (2008).

80. AEMET. *Atlas climático ibérico*. (Agencia Estatal de Meteorología, Ministerio de Medio Ambiente y Medio Rural y Marino, 2011)

81. Kellermann, J. (Ed.) *Flora of South Australia,* 5th edition. (Department of Environment, Water and Natural Resources, Government of South Australia, 2016).

82. Thackway, R., & Cresswell, I. D. *An Interim Biogeographic Regionalisation for Australia: a Framework for Establishing the National System of Reserves*, Version 4.0. (Australian Nature Conservation Agency, 1995).

83. Sanz-Elorza, M., Sánchez, E. D. D., & Vesperinas, E. S. Atlas de las plantas alóctonas invasoras en España (Organismo Autónomo Parques Nacionales, Madrid).

http://www.magrama.gob.es/es/biodiversidad/temas/inventarios-nacionales/c4_sinopsis_tcm7-21524.pdf) (2004).

84. Marticorena, C. & Quezada, M. Catálogo de la flora vascular de Chile. Gayana Botánica 42, 5–157 (1985).
85. Fuentes, N., Pauchard, A., Sánchez, P., Esquivel, J. & Marticorena, A. A new comprehensive database of alien plant species in Chile based on herbarium records. Biol. Invasions 15, 847-858 (2013).
86. Zuloaga, F. O., Morrone, O. & Belgrano, M. J. Catálogo de las plantas Vasculares del Cono Sur. http://www2.darwin.edu.ar/Proyectos/FloraArgentina/fa.htm (2008).
87. Castroviejo, S. Flora Iberica, vols. 1-15, 17-18, 20-21 (Real Jardín Botánico de Madrid, C.S.I.C., Madrid). http://www.floraiberica.org/ (1986-2015)
88. Tutin, T. et al. Flora Europaea, vols. 1–5. Cambridge: Cambridge University Press, Cambridge, 1964-1980).
89. Pyšek, P. & Jarošík, V. in Invasive plants: Ecological and agricultural aspects (ed Inderjit, S.) 77-96 (Springer Science & Business Media, New York, 2005).
90. Rejmánek, M. Invasive plants: approaches and predictions. Austral Ecol. 25, 497-506 (2000).
91. Cayuela, L., Granzow de la Cerda, I., Albuquerque, F. S. & Golicher, D. J. TAXONSTAND: an R package for species names standardisation in vegetation databases. Methods Ecol. Evol. 3, 1078–1083 (2012).
92. Pauchard, A., Cavieres, L., Bustamante, R., Becerra, P. & Rapoport, E. Increasing the understanding of plant invasions in Southern South America: first symposium on alien plant invasions in Chile. Biol. Invasions 6, 255 –257 (2004).
93. Beidleman, R. G. California's frontier naturalists (University of California Press, California, 2006).
94. Spencer, L. T. Naturalists of the Pacific shore: Early explorer/naturalists of California. Am. Zool. 26, 321-329 (1986).
95. Thunberg, C. P. Prodromus Plantarum Capensium (Edman, Uppsala, 1794).
96. Thunberg, C. P. Flora Capensis. (Edman, Uppsala, 1813).
97. Rourke, J. P. Plant systematics in South Africa: a brief historical overview, 1753–1953. Trans. Roy. Soc. S. Afr. 54, 179-190 (1999).
